# Phyto-fabricated iron oxide nanoparticles from *Phyllanthus acidus*: a magnetically recoverable nanoadsorbent for rapid Congo red detoxification

**DOI:** 10.1039/d6ra02105k

**Published:** 2026-04-21

**Authors:** Md. Ahad Mahmud Nahim, Asifur Rahman, Jamil Ahmed, Jannatul Naime, Md. Abu Rayhan Khan, Shofiur Rahman, Mahmoud A-Gawati, Habib Md. Ahsan

**Affiliations:** a Chemistry Discipline, Khulna University Khulna-9208 Bangladesh ahsanhru@chem.ku.ac.bd; b Graduate School of Life Science, Tohoku University 2-1-1 Katahira, Aoba Ku Sendai-980-8577 Japan; c Department of Chemistry, Mississippi State University USA; d King Abdullah Institute for Nanotechnology, King Saud University Riyadh 11451 Saudi Arabia mrahman1@ksu.edu.sa

## Abstract

This study reports the sustainable synthesis of phyto-fabricated magnetite iron oxide nanoparticles (IONPs) using *Phyllanthus acidus* leaf extract as a natural reducing and capping agent *via* a green co-precipitation route. The synthesized IONPs were extensively characterized; X-ray diffraction (XRD) confirmed crystallinity, and Fourier transform infrared spectroscopy (FTIR) identified key functional groups, such as Fe–O. Microscopic analysis (SEM/TEM) revealed polydispersed spherical particles (∼35 nm) capped with phytochemicals, while vibrating sample magnetometer (VSM) analysis demonstrated superparamagnetic behavior with a saturation magnetization of 60 emu per g. The nanoadsorbent exhibited exceptional performance in removing Congo Red (CR) dye, achieving a Langmuir monolayer capacity of 233.21 mg g^−1^ and over 95% removal efficiency within 80 minutes. Furthermore, the IONPs maintained 76.5% of their initial efficiency after five cycles, showcasing robust stability and reusability. By leveraging the adsorption efficiency of these nanoparticles, this eco-friendly fabrication route offers a scalable and cost-effective strategy for wastewater remediation and dye detoxification.

## Abstract 2

This study reports the sustainable synthesis of phyto-fabricated magnetite iron oxide nanoparticles (IONPs) using *Phyllanthus acidus* leaf extract as a natural reducing and capping agent *via* a green co-precipitation route.

## Graphical abstract

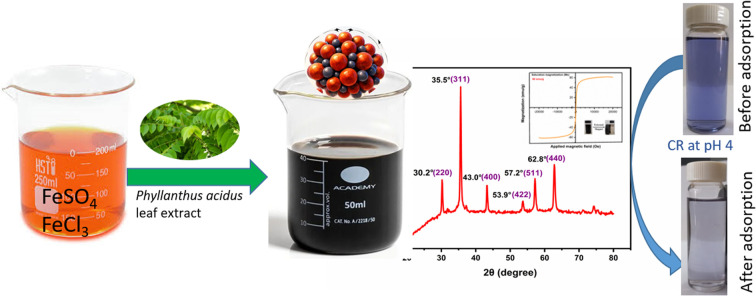

## Introduction

Groundwater contamination remains one of the most formidable challenges of the modern industrial era. Rapid population growth, uncontrolled urban expansion, and aggressive industrialization have led to the continuous discharge of diverse pollutants into aquatic ecosystems.^1^ Among these, synthetic organic dyes predominantly from the textile, rubber, and paper industries are of significant global concern due to their high solubility, chemical stability, and inherent resistance to conventional biodegradation.^2,3^ Even at trace concentrations, these persistent pollutants drastically impair water quality. They obstruct sunlight penetration and inhibit photosynthesis, thereby increasing biochemical oxygen demand (BOD) and potentially yielding mutagenic or carcinogenic intermediates during natural degradation processes.^4,5^ CR, a benzidine-based anionic azo dye, typifies this environmental threat. Its complex chromophoric structure not only imparts intense coloration to water and further blocks essential sunlight but also exhibits strong resistance to chemical oxidation.^6,7^ CR was selected as a representative pollutant due to its recalcitrant nature and its widespread use as a model anionic dye, providing a robust benchmark for evaluating the adsorption performance and practical applicability of the synthesized nanoparticles. This choice also enables direct comparison with existing literature while addressing its well-documented environmental and health risks, particularly its carcinogenic effects in aquatic ecosystems.^8^ While conventional treatment technologies such as coagulation–flocculation, membrane separation, and biological processes are widely deployed, they are often hindered by high operational costs, heavy energy demands, secondary sludge generation, and low efficacy against stable azo bonds.^9^ Consequently, there is an urgent need for sustainable alternatives. Adsorption and photocatalysis have emerged as premier alternatives due to their simplicity, cost-effectiveness, and ability to mineralize dyes into harmless byproducts like CO_2_ and H_2_O under visible light.^10,11^ In this context, nanoparticles (NPs) serve as superior catalysts and adsorbents. Their high surface-to-volume ratio facilitates rapid mass transfer and increased reactive site availability, leading to enhanced catalytic performance compared to bulk materials.^12,13^ While various transition metal oxides (ZnO, TiO_2_, CuO) are utilized in remediation, IONPs have gained prominence due to their exceptional biocompatibility, redox-active Fe^2+^/Fe^3+^ sites, and environmental stability. Magnetite (Fe_3_O_4_), in particular, is highly valued because its superparamagnetic nature allows for rapid recovery from aqueous media using an external magnetic field, eliminating the need for complex filtration and enabling easy reusability.^14–16^ Despite the effectiveness of IONPs, traditional physical and chemical synthesis methods often rely on toxic reducing agents, high temperatures, or hazardous solvents, which limit their green credentials and scalability.^17^ Conversely, plant-mediated green synthesis offers an eco-friendly and sustainable path. Plant extracts are naturally rich in polyphenols, flavonoids, and organic acids that act as both reducing agents and capping ligands. This dual mechanism prevents nanoparticle agglomeration and supports rapid nucleation under ambient conditions without the need for additional surfactants.^18–21^*Phyllanthus acidus* (Star Gooseberry), a plant native to Southeast Asia, is renowned for its high concentration of bioactive phytochemicals, including gallic acid, ellagic acid, and quercetin.^22,23^ Although these phytochemicals have been employed in the synthesis of various metallic nanoparticles, their application in the green synthesis of iron oxide nanoparticles remains largely unexplored, particularly in the context of dye adsorption and wastewater remediation. Addressing this gap, the present study introduces a one-pot green synthesis approach that eliminates the need for external surfactants or hazardous reducing agents. The novelty of this work lies in the fabrication of highly stable, superparamagnetic IONPs with enhanced adsorption efficiency and reusability, offering a sustainable green-to-green solution for industrial dye detoxification.

Therefore, the primary aim of this study is to develop and characterize phyto-fabricated IONPs using *Phyllanthus acidus* leaf extract as a dual-functional reducing and capping agent, and to systematically evaluate their efficiency, adsorption behavior, and reusability for the removal of Congo red dye from aqueous systems, thereby establishing a sustainable and environmentally benign strategy for wastewater remediation.

## Results and discussion

**Fig. 1:**
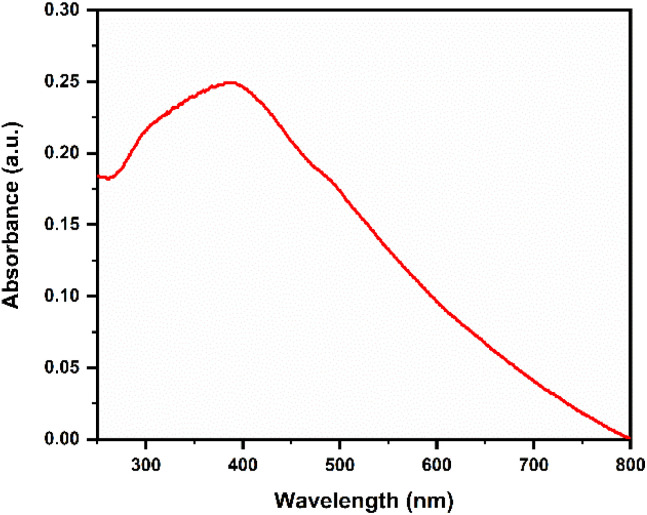
UV-visible spectrum of phyto-fabricated megnetite IONPs green-synthesized using *Phyllanthus acidus* leaf extract (condition 80 °C and 2 hours).

The synthesis of IONPs was initially monitored *via* UV-vis spectroscopy, a sensitive technique for detecting the bioreduction and stabilization of metal ions. The transition of the reaction mixture from a greenish-brown to a characteristic dark reddish-brown color change provided preliminary qualitative evidence of the Fe^3+^/Fe^2+^ reduction mediated by the bioactive phytochemicals present in the *Phyllanthus acidus* leaf extract. For quantitative spectroscopic analysis, a stock dispersion of the synthesized IONPs was prepared in deionized (DI) water and subjected to probe sonication to ensure a homogeneous, non-agglomerated particle distribution. The UV-vis absorption spectrum of the *Phyllanthus acidus*-mediated IONPs were recorded across a spectral range of 200–800 nm. The recorded spectra exhibited a prominent absorption band centered at approximately 391 nm, attributed to electronic transitions and characteristic surface resonance-like features of magnetite nanoparticles, confirming successful synthesis (Fig. 1).^24^

**Fig. 2:**
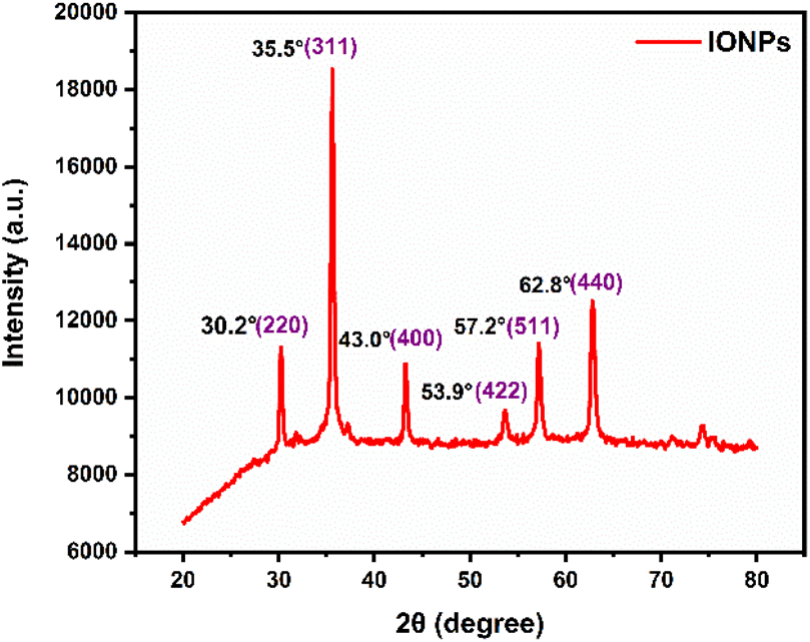
PXRD pattern of phyto-fabricated IONPs synthesized using *Phyllanthus acidus* leaf extract (condition 80° and 2 hours).

XRD was employed to characterize the crystalline structure and phase composition of the synthesized nanoparticles (NPs). As illustrated in Fig. 2, the green-synthesized IONPs exhibit six distinct diffraction peaks at 2*θ* values of approximately 30.33°, 35.65°, 43.31°, 53.72°, 57.24°, and 62.81°. These peaks correspond to the (220), (311), (400), (422), (511), and (440) Miller indices, respectively. The observed reflections align closely with the standard cubic spinel structure of iron oxide (JCPDS card no. 019-0629).^25^ This indexing indicates that the sample is predominantly composed of magnetite (Fe_3_O_4_), though a minor contribution from maghemite (γ-Fe_2_O_3_) cannot be entirely ruled out due to their structural similarities.^26^

The average crystallite size was calculated using the Scherrer equation:
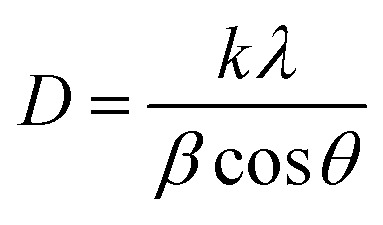
where *D* is the average crystallite diameter, *k* Scherrer constant (dimensionless shape factor, typically 1 for spherical particles). *λ* is the X-ray wavelength (0.1541 nm for Cu-Kα), *β* is the full width at half maximum (FWHM) of the diffraction peak, and *θ* is the corresponding Bragg angle (half of the 2*θ* value). Calculations across the indexed planes yielded crystallite sizes ranging from ≈12 nm. The consistency among these values suggests that the IONPs possess a high degree of crystalline uniformity and can be characterized as effectively monodisperse.

**Fig. 3:**
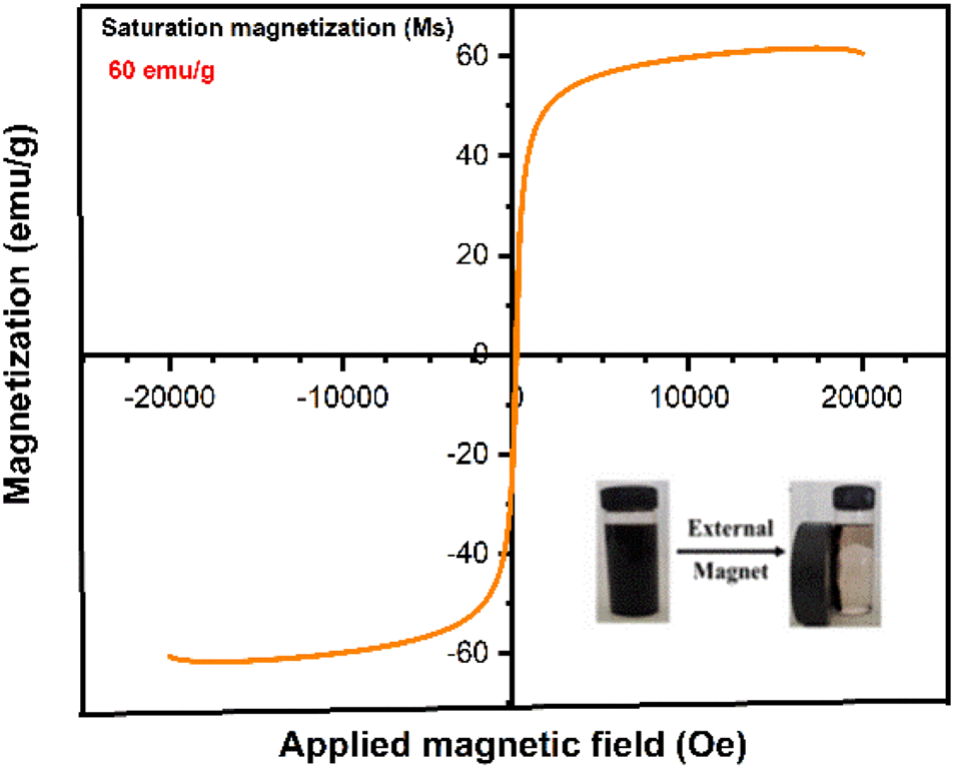
Optical image and VSM magnetization curve of phyto-fabricated IONPs synthesized using *Phyllanthus acidus* leaf extract (condition 80 °C and 2 hours).

The magnetic properties of the *Phyllanthus acidus* -mediated IONPs were evaluated at ambient temperature. As illustrated in the magnetization (*M–H*) curve in Fig. 3, the NPs exhibit a clear sigmoidal profile with negligible remanence (*M*_r_) and coercivity (*H*_c_). The absence of a hysteresis loop is the defining characteristic of superparamagnetic behavior, which occurs when the thermal energy at room temperature is sufficient to overcome the magnetic anisotropy energy of the individual nanoparticles. The saturation magnetization (*M*_s_) was measured at 60 emu per g. While this value is lower than that of bulk magnetite (92 emu per g), a phenomenon typically attributed to surface spin canting and the presence of a non-magnetic organic capping layer, it remains exceptionally high for green-synthesized IONPs.^27^ This strong magnetic responsiveness ensures that the nanoadsorbents can be rapidly and quantitatively isolated from aqueous media using a low-intensity external magnetic field, thereby facilitating ease of recovery and multiple reusability cycles without the need for energy-intensive filtration.

**Fig. 4:**
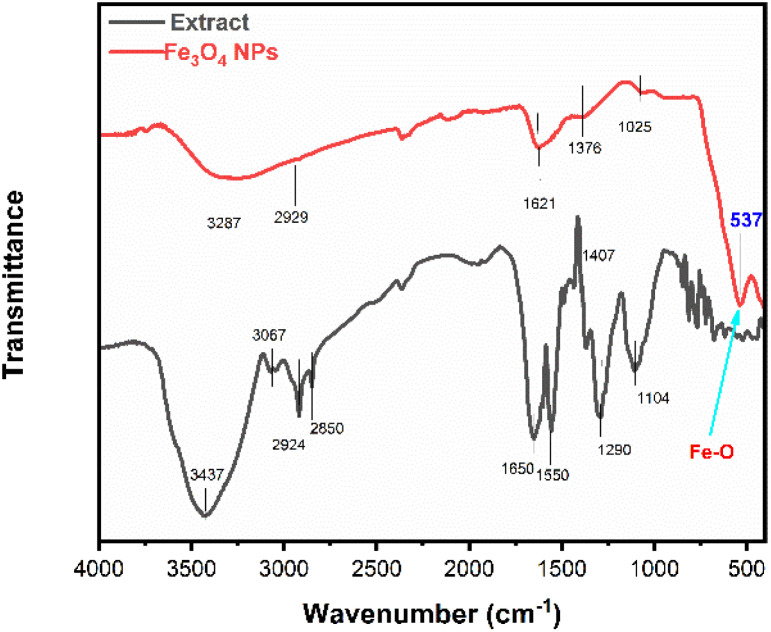
Comparative FTIR spectra of the solid content of *Phyllanthus acidus* leaf extract and IONPs synthesized using *Phyllanthus acidus* (condition 80 °C and 2 hours).

FTIR analysis was performed to elucidate the functional groups involved in the bioreduction, capping, and stabilization of the synthesized IONPs. Fig. 4 illustrates a comparative spectral analysis of the *Phyllanthus acidus* leaf extract and the resulting IONPs. The plant extract exhibited several characteristic absorption bands at 3437, 3067, 2924, 2850, 1650, 1290, and 1104 cm^−1^, reflecting a complex profile of phytochemical constituents. Specifically, the intense, broad peak at 3437 cm^−1^ is attributed to the O–H stretching vibrations of phenolic and polyphenolic compounds. The bands at 2924 and 2850 cm^−1^ correspond to the symmetric and asymmetric C–H stretching of aliphatic chains.^28^ Furthermore, the absorption at 1650 cm^−1^ represents the C=O stretching of carboxylic acids or flavonoids, while the peak at 1104 cm^−1^ arises from C–O stretching vibrations within aromatic structures.^29^ Additionally, the week absorption at 2300 cm^−1^ due to the atmospheric CO_2_.^30^ Upon the formation of IONPs, significant spectral shifts and intensity variations were observed, indicating the chemical interaction between phytochemicals and the iron precursors. The O–H stretching band shifted from 3437 cm^−1^ to a lower wavenumber of 3287 cm^−1^, suggesting the active participation of hydroxyl groups in the reduction of Fe^3+^/Fe^2+^ ions. Moreover, the shift in the C=O stretching frequency (1650 to1590 cm^−1^) and the C–O vibration (1104 to 1128 cm^−1^) indicates that carboxylate and phenolic groups served as capping agents, facilitating the steric stabilization of the nanoparticle surfaces. Crucially, a prominent new absorption band appeared at 537 cm^−1^ in the IONP spectrum. This peak is the fingerprint of Fe–O lattice vibrations in the octahedral and tetrahedral sites of the magnetite structure, providing definitive evidence for the successful fabrication of iron oxide nanoparticles.^27,31^

**Fig. 5:**
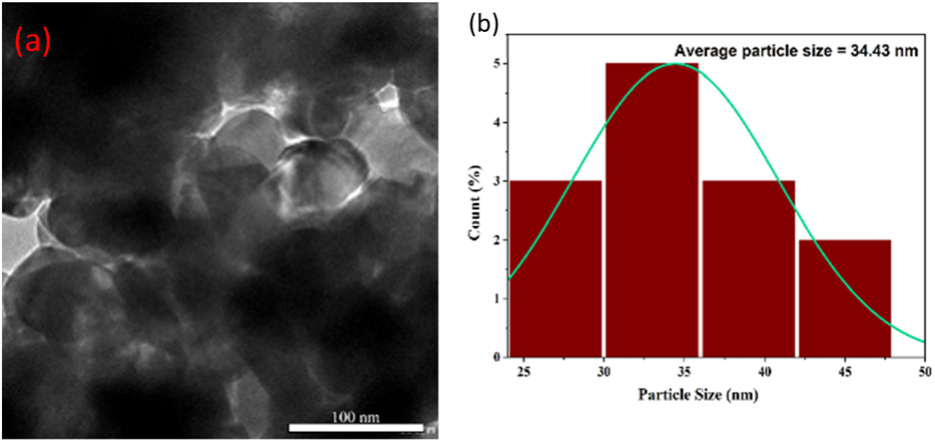
(a) TEM and (b) corresponding particle size distribution histogram of magnetite IONPs synthesized using *Phyllanthus acidus* (condition 80 °C and 2 hours).

The morphological characteristics and size distribution of the *Phyllanthus acidus* -mediated IONPs were investigated *via* High-Resolution Transmission Electron Microscopy (HR-TEM), as illustrated in Fig. 5(a). The micrographs reveal that the nanoparticles possess a predominantly quasi-spherical to irregularly polyhedral geometry. A distinct contrast variation is observed across the nanostructures: the particles exhibit highly electron-dense cores, often surrounded by lighter, translucent peripheral fringes or halos. This core-shell-like architecture suggests the presence of a well-defined crystalline iron oxide nucleus encapsulated by a thin layer of plant-derived organic capping agents, which facilitates steric stabilization.^26^

The nanoparticles appear as loose, polydisperse agglomerates rather than isolated grains. This clustering is characteristic of green-synthesized magnetic nanomaterials, driven by both the high surface energy of the particles and inherent magneto-dipolar attractions. Similar morphological trends have been documented in IONPs synthesized using Myristica fragrans and Egeria extracts, which typically yield irregularly shaped, clustered grains.^32,33^

The particle size distribution was quantified using ImageJ software by measuring over 13 individual nanoparticles from the HRTEM images. As shown in the histogram in Fig. 5(b), the synthesized IONPs exhibit an average particle size of 34.92 nm. This result aligns with the sub-100 nm range typical for biosynthesized Fe_3_O_4_ systems. Interestingly, while a previous study utilizing *Phyllanthus acidus* extract reported the formation of rod-like structures (∼19 nm),^34^ our specific co-precipitation conditions favored the growth of equiaxed, quasi-spherical grains. This morphological divergence highlights the critical influence of precursor concentration, pH, and extract chemistry on the final nanostructure. Despite the polydispersity, the dense cores and lattice fringes observed confirm the successful fabrication of crystalline magnetite/maghemite nanoparticles.

**Fig. 6:**
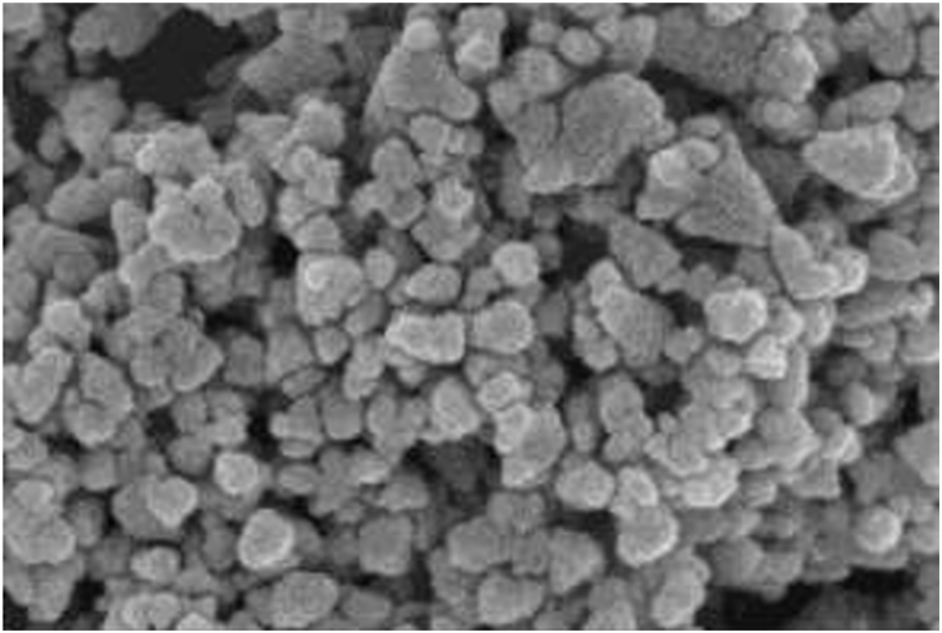
FE-SEM image of magnetite IONPs synthesized using *Phyllanthus acidus* (condition 80 °C and 2 hours).

The surface topography and morphological features of the bio-synthesized IONPs were investigated using Field Emission Scanning Electron Microscopy (FE-SEM). Unlike conventional SEM, FE-SEM utilizes a high-brightness electron source to provide superior spatial resolution, capturing intricate details of the specimen's surface topography and grain boundaries. As illustrated in the micrographs in Fig. 6, the IONPs exhibit a predominantly spherical morphology with a high degree of structural uniformity. The particles appear as discrete, homogeneous granular spheres with minimal elongation or shape anisotropy. These results are in excellent agreement with literature reports on plant-mediated Fe_3_O_4_ systems, which frequently describe the formation of regularly shaped, narrowly distributed nanoscale spheres under controlled green synthesis conditions. At higher magnifications, the presence of interparticle clustering becomes evident. The observed aggregation results from several competing physicochemical drivers: long-range magnetic dipole–dipole interactions, van der Waals attraction, and the inherent tendency to minimize high surface free energy. These forces promote the formation of small agglomerates and fused clusters, which is a well-documented phenomenon in magnetic ferrites such as NiFe_2_O_4_ and Fe_3_O_4_. This localized overlapping and clustering account for the slight polydispersity observed in the high-size tail of the particle distribution histogram. Despite this minor agglomeration, the FE-SEM analysis confirms that the *Phyllanthus acidus* extract successfully acts as a capping agent, maintaining a relatively stable, equiaxed particle morphology suitable for high-surface-area applications like dye adsorption.

**Fig. 7:**
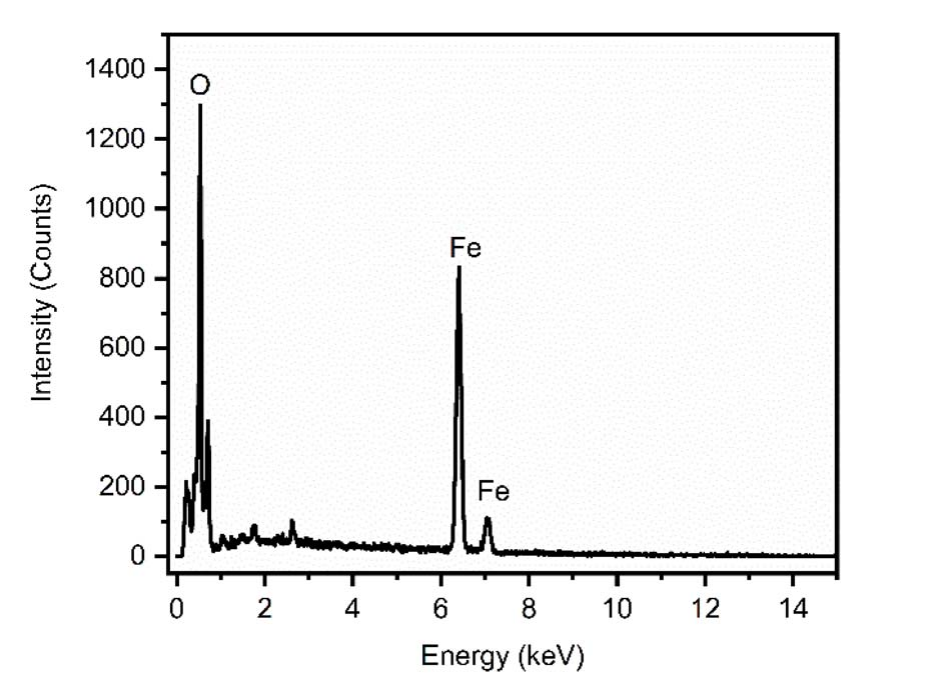
EDX spectrum of IONPs synthesized using using *Phyllanthus acidus* (condition 80 °C and 2 hours).

The elemental composition and purity of the synthesized IONPs were evaluated using Energy Dispersive X-ray (EDX) spectroscopy. The representative EDX spectrum, illustrated in Fig. 7, confirms the successful formation of iron oxide nanoparticles through the distinct signals of Fe (69%) and O (24%) and rest of element of C and N presence of residual organic moieties from the *Phyllanthus acidus* leaf extract, which likely act as capping and stabilizing agents on the nanoparticle surface.^35^

## Adsorption studies

### Effect of solution pH

**Fig. 8:**
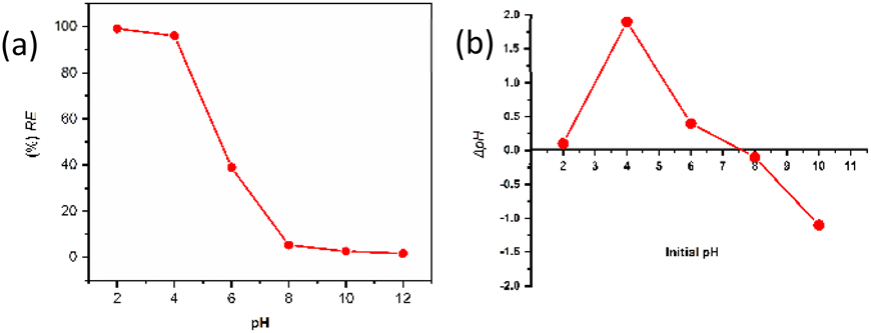
Adsorption studies of CR using phyto-fabricated magnetite IONPs (a) effect of pH on adsorption efficiency (*C*_0_ = 25 mg L^−1^, *w* = 15 mg and *V* = 100 mL), and (b) point zero charge (pH_pzc_) determination curve for IONPs.

The adsorption of CR onto the synthesized IONPs exhibited a pronounced dependence on solution pH, which governs both the adsorbent surface charge and the ionization state of the dye molecules. As illustrated in Fig. 8(a), the CR removal efficiency (*C*_0_ = 25 ppm, adsorbent dose = 15 mg) decreased significantly as the pH transitioned from acidic to alkaline conditions. To elucidate this behavior, the point of zero charge (pH_pzc_) of the IONPs was determined using the ΔpH method. The intersection of the ΔpH *vs.* ΔpH_initial_ curve with the *x*-axis indicates a pH_pzc_ of approximately 7.5. At pH < pH_pzc_, the nanoparticle surface undergoes protonation, resulting in a net positive surface charge (Fe–OH^2+^) and the particles repel each other, lowering the particle size.^36^ The increase in surface area of smaller particles can facilitate the dye removal efficiency. On the other hand, CR dye remains negatively charged above pH 3 due to sulfonate groups (SO_3_^−^).^37^ An electrostatic attraction between IONPs and CR, as well as the increased surface area, results in significant removal efficiency as found in Fig. 8(b). Conversely, at pH > pH_pzc_, the deprotonation of surface hydroxyl groups leads to a negatively charged surface (Fe–O^−^). The resulting coulombic repulsion between the negatively charged IONPs surface and the anionic dye molecules causes a dramatic decline in adsorption capacity. The maximum removal efficiency observed at pH ≈ 4 confirms that electrostatic forces are the primary drivers of the adsorption mechanism. These results underscore that optimal remediation is achieved in slightly acidic environments where the positive surface potential of the phyto-fabricated IONPs maximizes the attraction to the target anionic pollutant. In contrast, for more alkaline solutions, the loss of attractive forces explains the observed decline in removal efficiency, which is consistent with other reports.^38,39^

### Effect of initial contact time

**Fig. 9:**
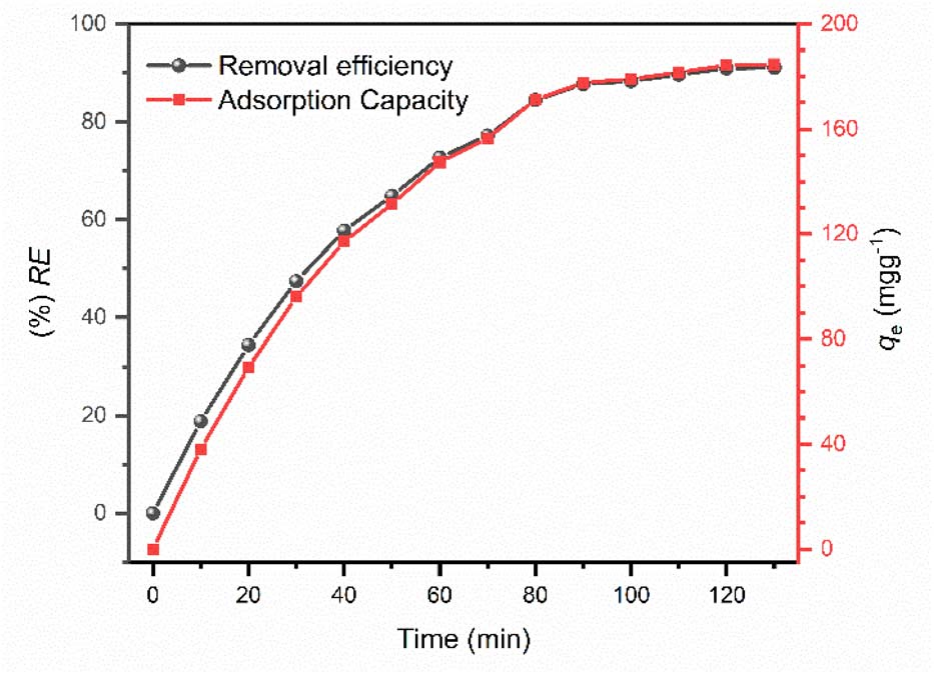
Time-dependent adsorption of CR onto IONPs. Red line: adsorption capacity and Black line: removal efficiency (condition *C*_0_ = 25 mg L^−1^, *w* = 15 mg, *V* = 100 mL and pH = 4).

The influence of contact time on the removal of CR was investigated over a duration of 130 min to determine the time required to reach adsorption equilibrium. As illustrated in Fig. 9, the characteristic absorption bands of CR progressively diminished as contact time increased, providing a spectral signature of the adsorption process. The adsorption process exhibited a distinctive two-stage kinetic profile. During the initial 80 min, a rapid decline in absorbance was observed, corresponding to a high rate of dye sequestration. This primary stage is attributed to the abundance of vacant active sites on the IONP surface and the high concentration gradient between the bulk solution and the adsorbent interface, which facilitates rapid external mass transfer.

Beyond 80 min, the rate of adsorption significantly decelerated, with the UV-vis spectra approaching a plateau. This transition signifies that the system is entering the equilibrium phase, where the remaining active sites become increasingly occupied, and the intra-particle diffusion resistance becomes the rate-limiting factor. The stabilization of the absorbance bands after 80 min indicates that the IONP surface has reached a saturation state, achieving maximum dye uptake under the specified conditions. Consequently, 80 min was established as the optimal equilibrium time for the IONPs-CR system, as further contact duration yielded negligible improvements in removal efficiency. This rapid kinetic response underscores the high affinity of the bio-engineered magnetite nanoparticles for anionic azo dyes.^40^

### Effect of adsorbent dosage

**Fig. 10:**
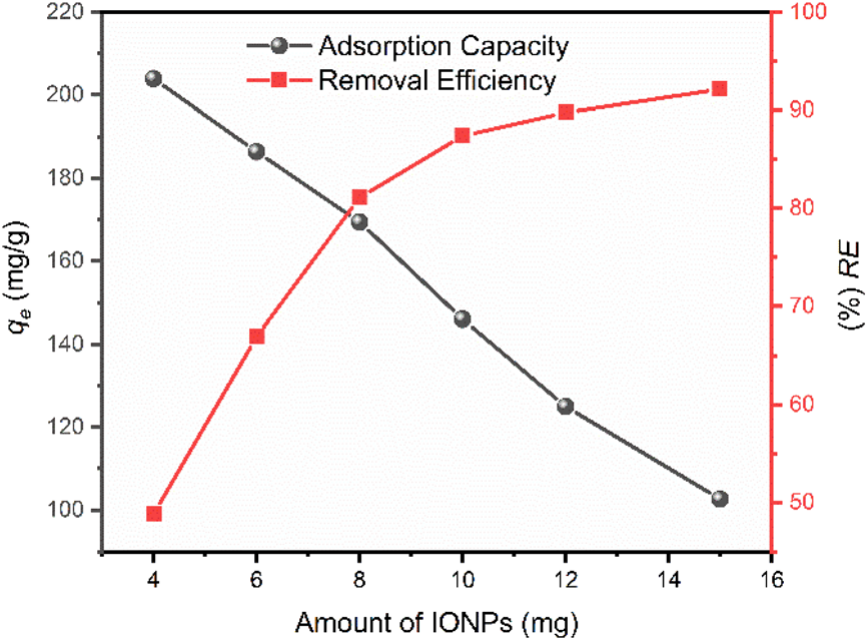
Dosage effect of IONPs on the adsorption process (*C*_CR_ = 25 mg L^−1^, *V* = 100 mL, *w* = 4–15 mg, and pH = 4).

The influence of nanoadsorbent dosage on the sequestration of CR was investigated by varying the IONP mass from 4 mg to 15 mg at a constant initial dye concentration (25 mg L^−1^). As illustrated in Fig. 10, increasing the adsorbent dose led to a significant enhancement in the overall dye removal efficiency, rising from approximately 48% to 95%. This trend is directly attributed to the increased total surface area and the resulting abundance of available active binding sites, which increase the probability of collision and capture of dye molecules from the bulk solution.^41^ Conversely, the equilibrium adsorption capacity (*q*_e_) exhibited an inverse relationship, declining from 210 to 105 mg g^−1^ as the dosage increased. This phenomenon occurs because the ratio of dye molecules to available adsorption sites decreases as the dose rises. At higher dosages, the total number of adsorption sites exceeds the number of adsorbate molecules present in the fixed volume. Consequently, many active sites remain unsaturated or idle, resulting in lower per-unit mass uptake. This dilution effect of the adsorption capacity is a common characteristic in batch studies, where the system reaches a plateau in removal efficiency while the individual capacity of the adsorbent is underutilized due to the saturation of the available dye molecules rather than the sites themselves.^42^ Therefore, an optimal dosage is critical to balance high removal efficiency with cost-effective adsorbent utilization.

### Influence of initial concentration

The effect of initial CR concentration on the adsorption performance was evaluated by varying the concentration from 5 to 25 mg L^−1^ at a constant adsorbent dose (15 mg) and optimized pH (pH 4).

**Fig. 11:**
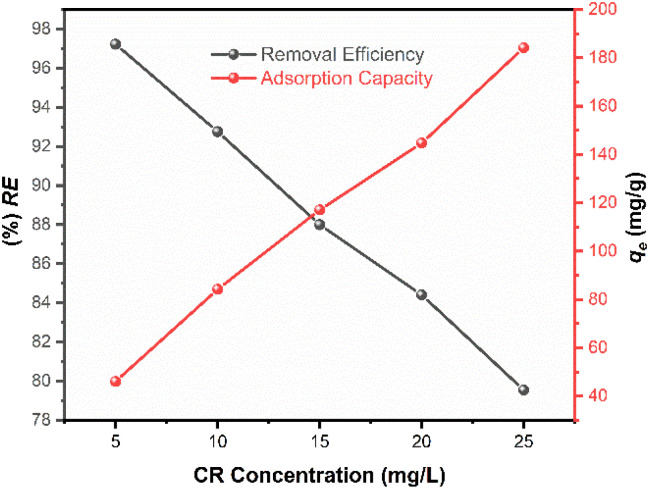
Effect of the initial CR concentration on the adsorption process (*C*_CR_ = 5–25 mg L^−1^, *V* = 100 mL, *w* = 15 mg, and pH = 4).

As illustrated in Fig. 11, an increase in the initial CR concentration resulted in a decrease in the overall removal efficiency from 98% to 81%. This trend is attributed to the finite number of active binding sites available on a fixed mass of IONPs.

At lower concentrations, the ratio of available surface sites to dye molecules is high, allowing for near-quantitative sequestration of the adsorbate. However, as the concentration increases, these specific sites become increasingly occupied, leading to site saturation and a subsequent reduction in the percentage of dye removed.Conversely, the equilibrium adsorption capacity (*q*_e_) demonstrated a positive correlation with the initial dye concentration. This phenomenon is driven by the enhanced concentration gradient at the solid–liquid interface, which provides a significantly stronger thermodynamic driving force to overcome the mass transfer resistance between the aqueous phase and the adsorbent surface. A higher concentration of CR molecules increases the frequency of collisions with the IONP surface, resulting in a greater mass of dye being adsorbed per unit mass of the catalyst. Consequently, while higher initial loadings lead to a reduction in the percentage removal due to the exhaustion of surface sites, they maximize the utilization of the adsorbent's absolute capacity.

## Adsorption kinetics

The adsorption kinetics of CR onto the biosynthesized IONPs were investigated at an initial concentration of 25 mg L^−1^ to elucidate the rate-controlling steps of the process. As shown in Fig. 9, the CR uptake was exceptionally rapid during the first 30 minutes, followed by a gradual deceleration as the system approached equilibrium at approximately 120 minutes.

**Fig. 12:**
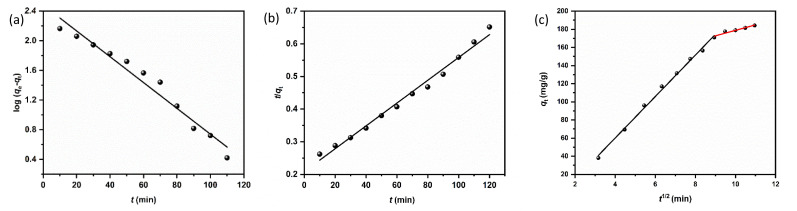
(a) Pseudo first order, (b) Pseudo second order, and (c) IPD kinetic model for the adsorption of CR using synthesized IONPs (condition *C*_0_ = 25 mg L^−1^, *w* = 15 mg, *V* = 100 mL and pH = 4).

At this stage, a removal efficiency of 90% was achieved, with an experimental adsorption capacity (*q*_exp_) of 184 mg g^−1^. To mathematically describe the adsorption rate, the time-dependent data were fitted to the pseudo-first-order (PFO), pseudo-second-order (PSO), and Weber–Morris intraparticle diffusion (IPD) models (Fig. 12). The calculated kinetic parameters are summarized in Table 1. The pseudo-second-order model exhibited a superior fit to the experimental data, characterized by a higher correlation coefficient (*R*^2^ = 0.99) and a theoretical equilibrium capacity (*q*_cal_ = 186 mg g^−1^) that closely aligns with the experimental value (184 mg g^−1^). In contrast, the PFO model significantly overestimated the capacity (201 mg g^−1^), indicating poor reliability. The dominance of the PSO model suggests a strong adsorbent–adsorbate interaction, which is the rate-limiting mechanism, is governed by chemisorption, involving the sharing or exchange of electrons between the IONP surface functional groups and the dye molecules.^35^Fig. 12(c) shows multi-linearity, where the initial steeper line (*t*^1/2^ < 9 min^1/2^) can be assigned to the diffusion of dye molecules from the bulk solution onto the surface of the IONPs. The second line (*t*^1/2^ > 9 min^1/2^) can be attributed to the diffusion of surface-adsorbed dye molecules into the internal pores. This diffusion is relatively slow, causing a slow adsorption rate. According to Wang *et al.*, the piecewise functions *q*_*t*_ = *k*_1_*t*^1/2^ (0 ≤ *t* ≤ *t*_1_) is due to the first stage of diffusion and *q*_*t*_ − *q*_*t*=t_1__ = *k*_2_(*t* − *t*_1_)^1/2^ (*t*_1_ < *t* ≤ *t*_2_) is due to the second stage.^43^

**Table 1:** Obtained data from kinetic models for CR adsorption

Models and equations	Parameters			
**Pseudo-first order**	***k***_1_ (×10^−2^ min^−1^)	***q*** ^**cal**^_**e**_ **(mg g^−^** ^**1**^ **)**	***q*** ^**exp**^_**e**_ **(mg g^−^** ^**1**^ **)**	***R*** ^**2**^
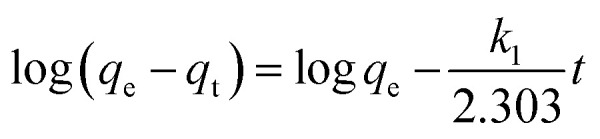	4.0	201	184.21	0.965
**Pseudo-second order**	*k*_2_ **(×10^−^** ^**5**^ **g mg^−^** ^**1**^ **min^−1^)**	***q*** ^**cal**^_**e**_ **(mg g^−^** ^**1**^ **)**	***q*** ^**exp**^_**e**_ **(mg g^−^** ^**1**^ **)**	***R*** ^**2**^
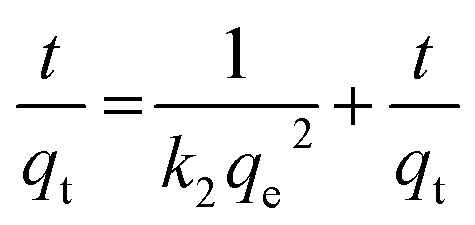	5.83	186.4	184.21	0.987
**Intraparticle diffusion**	*k*_dif_ **(mg (g^−^** ^**1**^ **min** ^**1/2**^ **))**	***R***_**i**_	***C* (mg g^−^** ^**1**^ **)**	***R*** ^**2**^
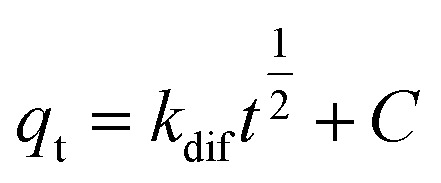	19.232	1.058	10.72	0.966
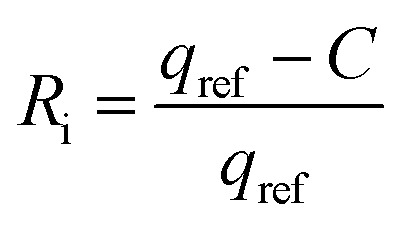				

Here *q*_*t*_ is the adsorption capacity at time *t*, *k*_1_ and *k*_2_ are the intraparticle diffusion rate constant. Greater values indicates the faster diffusion.

## Adsorption isotherm studies

**Fig. 13:**
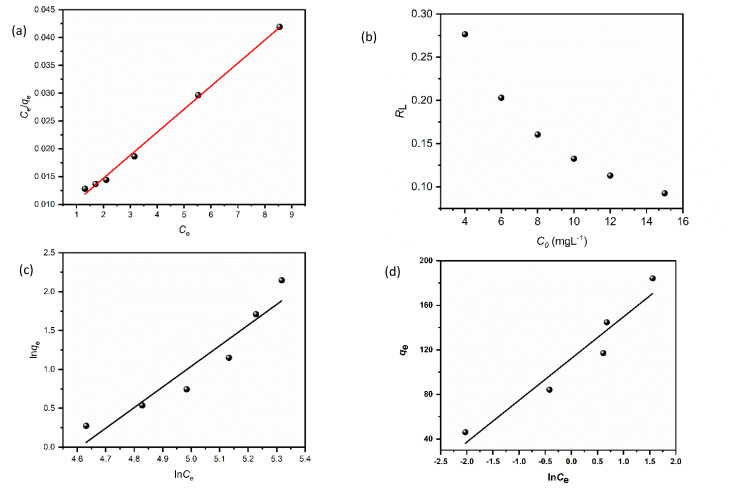
(a) Langmuir, (b) *R*_L_*vs C*_0_, (c) Freundlich, and (d) Temkin isotherm model for adsorption analysis of CR by synthesized IONPs at pH 4.

The equilibrium adsorption of CR onto biosynthesized IONPs was evaluated using the Langmuir, Freundlich, and Temkin isotherm models (Fig. 13). The calculated parameters, which provide insight into the surface properties and affinity of the adsorbent, are summarized in the Table 3.

The Langmuir model provided the most accurate fit for the experimental data, yielding an exceptionally high correlation coefficient (*R*^2^ = 0.9966). This suggests that the adsorption process is dominated by monolayer coverage onto a surface with a finite number of identical, energetically equivalent sites.^44^ The maximum adsorption capacity (*Q*_m_) is approximately 233.21 mg g^−1^. The separation factor (*R*_L_) values ranged from 0.03 to 0.16 for initial concentrations of 5–25 mg L^−1^. Since 0 < *R*_L_ < 1, as expected from pseudo second order fit, the adsorption process is confirmed to be highly favourable. Incontrast, the Freundlich (*R*^2^ = 0.9062) and Temkin (*R*^2^ = 0.9288) models showed lower correlations. While the Freundlich intensity parameter (*n* ≈ 2.57) indicates favorable uptake (*n* > 1) and degree of surface heterogeneity, the superior fit of the Langmuir model implies that the IONPs behave primarily as a homogeneous system under these conditions.^45^

To further evaluate the effectiveness and practical significance of the present phyto-fabricated IONPs, a comparative analysis with previously reported iron-based nanoadsorbents for CR removal is presented in Table 2. Various synthesis routes, including hydrothermal, chemical reduction, precipitation, and plant-mediated green synthesis, have been reported in the literature. However, these systems often suffer from limitations such as longer equilibrium times, higher adsorbent dosages, moderate removal efficiencies, or lack of kinetic and isotherm validation.

**Table 2:** Comparative performance of iron-based nanoadsorbents for CR removal under different synthesis routes and operational conditions

No.	IONP synthesis (method)	IONP dose	CR initial conc.	Contact time	pH	Removal (%)	Kinetics	Isotherm	Study (Ref.)
1	Hydrothermal method	1.0 g L^−1^	20 mg L^−1^	180 min (equilibrium ≈ 30 min)	7 (neutral)	>90%	Not modeled	Langmuir adsorption (*R*^2^ ≈ 0.99)	46
2	Hydrothermal method	10 mg in 20 mL (500 mg L^−1^)	20 mg L^−1^	120 min	∼6–7 (natural)	≈100% adsorbed (dark) after 120 min	Not modeled	Not analyzed	47
3	Precipitation method	2 mg in 200 mL (10 mg L^−1^)	25 mg L^−1^	60 min	6.5	72.99% (60 min)	Not modeled	Not analyzed	48
4	Phyto-assisted co-precipitation using *Acacia jacquemontii* extract	(Not specified)	(Not specified)	60 min	pH opt. acidic (max CR removal at acidic pH)	47.5% (60 min, at acidic pH)	Not analyzed	Not analyzed	49
5	Green synthesis *via* aloe *barbadensis* and *Camellia sinensis* extracts (Fe_2_O_3_ NPs)	(Not specified)	∼20 mg L^−1^	∼40–45 min	5 (optimum for CR)	∼75–80% (optimum ∼45 min)	Pseudo-second-order kinetics (*R*^2^ ∼0.997)	Langmuir & Elovich isotherms	50
6	Green synthesis with *Plantago lanceolata* leaf extract	15 mg/10 mL (1500 mg L^−1^)	50 mg L^−1^	180 min	8 (optimal)	99% (with sunlight + H_2_O_2_)	Pseudo-second-order kinetics	Langmuir isotherm (*R*^2^ ∼0.96)	51
7	S1 (bcc Fe^0^): FeCl_2_·4H_2_O reduced by NaBH_4_ in 80% ethanol–water; borohydride poured rapidly under stirring.	10 mg	50–150 mg L^−1^	Equilibrium: 10 h; 75% removed in 15 min	Neutral	∼75% in 15 min; ∼98% (1st cycle)	Pseudo-2nd order	Langmuir isotherm (*R*^2^ = 0.998)	52
	S2 (amorphous Fe^0^): Same method; NaBH_4_ added dropwise (1 drop/2 s)	10 mg	50–150 mg L^−1^	Equilibrium: 10 h; 85% removed in 15 min	Neutral (isotherm); pH 2–13 tested	85% in 15 min; 93% at pH 2; 13% at pH 13	Pseudo-2nd order	Langmuir isotherm (*R*^2^ = 0.996)	
8	Green synthesis with *Phyllanthus acidus* leaf extract	15 mg in 100 mL	25 mg L^−1^	∼80 min	4 (optimum for CR)	∼98%	Pseudo-second-order	Langmuir isotherm	This work

In contrast, the IONPs synthesized in this study exhibit superior adsorption performance, achieving approximately 98% removal of CR at a relatively low adsorbent dose (15 mg per 100 mL), shorter equilibrium time (∼80 min), and under mildly acidic conditions (pH 4). Furthermore, the adsorption process follows pseudo-second-order kinetics and fits well with the Langmuir isotherm model, indicating a chemisorption-driven monolayer adsorption mechanism. Compared to previously reported systems, the present study demonstrates a favorable balance between efficiency, operational conditions, and sustainability, highlighting the effectiveness of *Phyllanthus acidus*-mediated green synthesis for wastewater remediation applications.

### Proposed adsorption mechanism

**Fig. 14:**
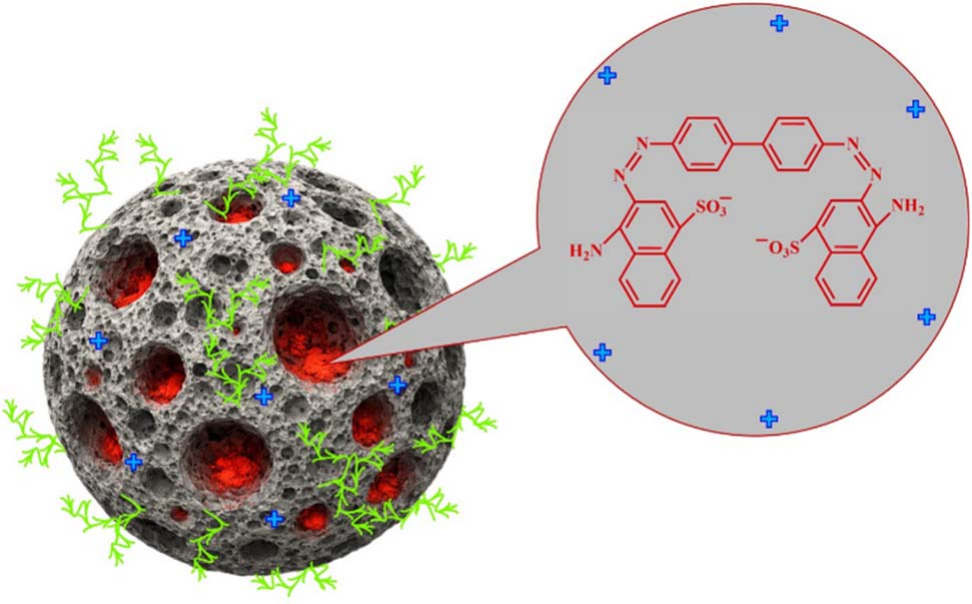
Schematic representation of the proposed adsorption mechanism of CR dye adsorption on IONPs.

The enhanced adsorption capacity observed at the experimental pH 4 can be attributed to a synergistic effect of electrostatic and molecular interactions. At pH 4 (below the point of zero charge for IONPs), the nanoparticle surface is protonated and carries a positive charge. This facilitates a strong electrostatic draw toward the negatively charged sulfonate (–SO_3_^−^) groups of the CR molecules. On the other hand, residual phytochemicals from the biosynthesis process (*e.g.*, phenolic –OH or –COOH groups) on the IONP surface act as donors/acceptors for hydrogen bonding with the amine (–NH_2_) moieties of CR (Fig. 14).

**Table 3:** Comparison of adsorption isotherm constants and statistical fit parameters for CR removal by IONPs

Models and equations	Constant	
**Langmuir**	*Q*_m_ **(mg g^−^** ^**1**^ **)**	233.21
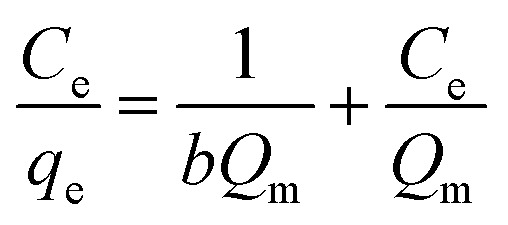	*R*_L_ = *C*_0_ = 5 mg L^−1^	0.1639
	= 10	0.08
	= 15	0.0613
	= 20	0.0467
	= 25	0.0377
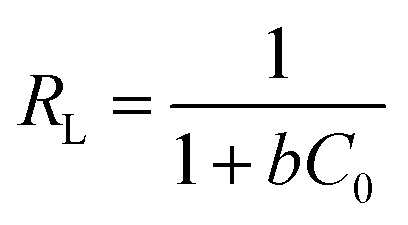	***R*** ^**2**^	**0.9966**
**Freundlich**	*n*	2.570
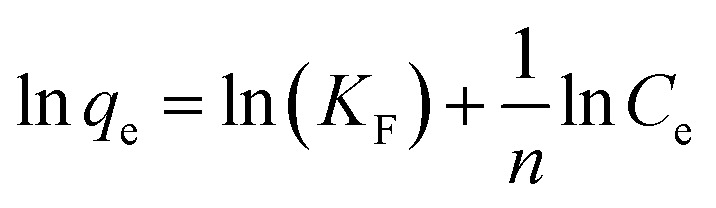	*K*_F_ (mg g^−1^(L mg^−1^)^1/*n*^)	100.71
	***R*** ^**2**^	**0.9062**
**Temkin**	^*b*^T	66.06
*q*_e_ = *B* ln*K*_T_ + *B* ln*C*_e_	*A*_T_(Lg^−1^)	19.88
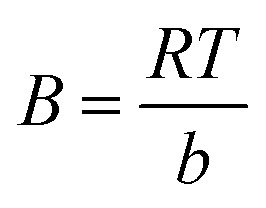	*B*(J mol^−1^)	37.51
	***R*** ^**2**^	**0.9288**

Ultimately, the data suggest that CR removal is a chemisorption-dominated process facilitated by a homogeneous monolayer, supported by the strong correlation with the Langmuir isotherm.^44,45,53^

### Reusability and stability studies

**Fig. 15:**
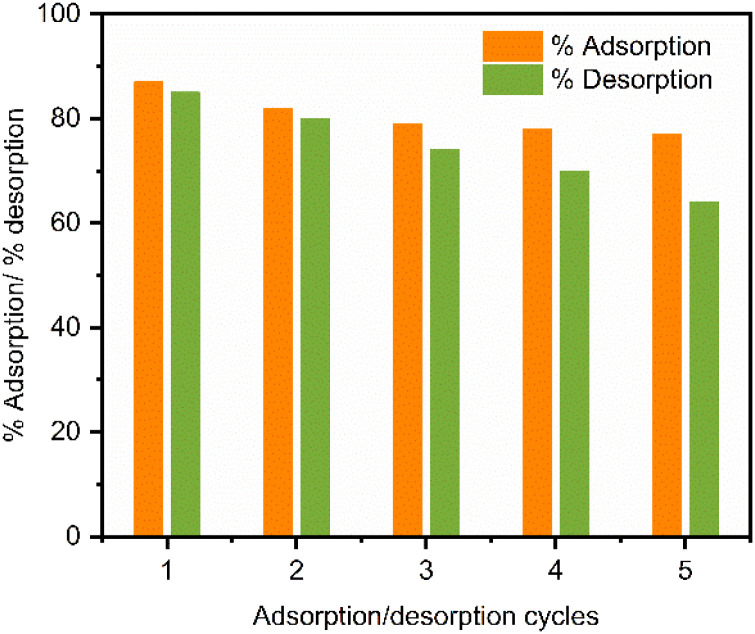
Reusability profile of IONPs for CR adsorption and desorption over five consecutive regeneration cycles.

The practical applicability and economic viability of the synthesized IONPs were evaluated through five consecutive adsorption–desorption cycles. Each adsorption cycle was conducted using 100 mL of a 25 mg L^−1^ CR solution and a 15 mg nanoadsorbent dose at an optimized pH of 4, and a desorption cycle was performed at pH = 12 using hydrochloric acid and sodium hydroxide to maintain the pH. As illustrated in Fig. 15, the CR removal efficiency exhibited a marginal decline, decreasing from 86.9% in the initial cycle to 76.5% by the conclusion of the fifth cycle, whereas the quantities of CR desorption varied within the same cycle from 84 to 64%. This gradual reduction in performance can be attributed to two primary factors.

(1) Partial desorption of dye, causing a saturation effect on the active sites over time, and (2) minor mechanical loss of IONPs during the sequential washing, centrifugation, and magnetic recovery stages, which effectively reduces the total adsorbent mass available for the reaction.^35^

**Fig. 16:**
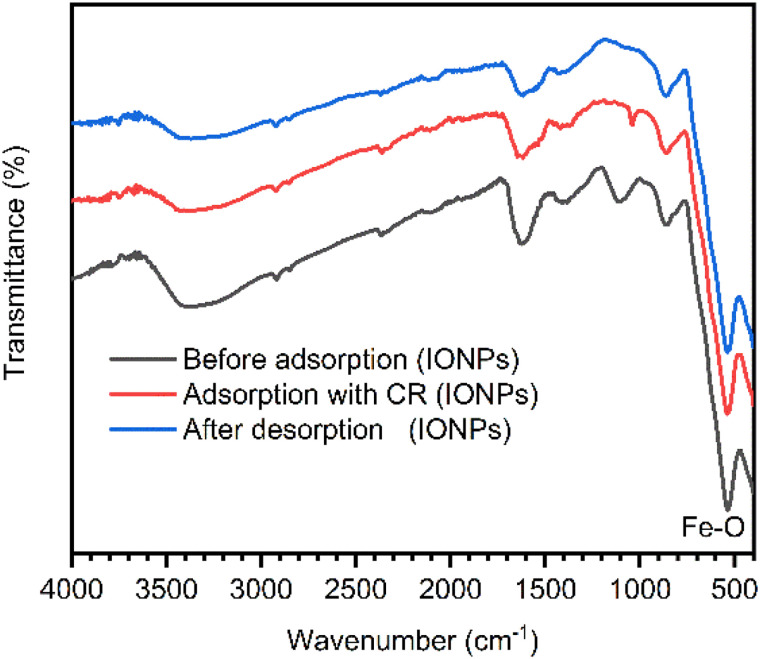
FTIR spectra of IONPs before adsorption, after adsorption of CR dye and after desorption.

Despite such a slight decrease in efficiency, the IONPs retained a substantial proportion of their original adsorption capacity across five cycles. This indicates the good stability and reusability of the catalyst, underscoring its promise as a potential recyclable adsorbent for repeated use in dye-contaminated wastewater treatment systems. Additionally, the stability of the IONPs following the adsorption–desorption cycle was confirmed using FTIR analysis (Fig. 16). A comparison of the spectra before and after demonstrated that the typical absorption bands, especially the Fe–O stretching vibration at approximately 536 cm^−1^, remained almost identical with respect to position and intensity.^54^ These results indicate the iron oxide structure is robust yet remains stable throughout the adsorption–desorption process.

## Experimental

### Materials and methods

#### Chemicals and reagents

All chemicals were of analytical reagent grade and used as received. Sodium hydroxide (NaOH), ferrous sulfate heptahydrate (FeSO_4_·7H_2_O), hydrochloric acid (HCl), ferric chloride (FeCl_3_), and Congo red (CR) dye were obtained from Merck (India). Deionized (DI) water obtained from a Milli-Q purification system was used throughout the study for solution preparation, washing, dilution, and adsorption experiments.

### Instrumentation and characterization

The optical properties of the synthesized materials were examined using a SHIMADZU UV-1900i UV-visible spectrophotometer in the wavelength range of 200–800 nm using a 1 cm quartz cuvette. Fourier transform infrared (FTIR) spectra were recorded on a SHIMADZU IRSpirit spectrophotometer in the range of 4000–400 cm^−1^ at a spectral resolution of 4 cm^−1^ using 32 scans per sample. Samples were prepared as KBr pellets. The crystalline structure of the synthesized nanoparticles was analyzed by powder X-ray diffraction (PXRD) using a Rigaku SmartLab diffractometer equipped with Cu-Kα radiation (*λ* = 0.1541 nm), operated at 40 kV and 30 mA. Diffraction data were collected over a 2*θ* range of 10–80° with a step size of 0.02° and a scan rate of 2° min^−1^. Surface morphology and elemental composition were investigated using a Hitachi SU-8000 field-emission scanning electron microscope (FE-SEM) coupled with EDX at accelerating voltages of 10 and 15 kV. Before FE-SEM observation, the dried powder samples were mounted on carbon tape and lightly sputter-coated with gold to minimize charging. Detailed particle morphology and size distribution were further examined using a JEOL TEM-2100F field-emission transmission electron microscope operated at 200 kV. For TEM analysis, the nanoparticles were dispersed in ethanol, sonicated for 10 min, drop-cast onto a carbon-coated copper grid, and dried under ambient conditions. Magnetic measurements were carried out at room temperature using a VSM 8604 vibrating sample magnetometer (Lake Shore Cryotronics Inc., USA) in an applied magnetic field range of −15 to +15 *k* Oe.

### Preparation of plant extract

Fresh leaves of *Phyllanthus acidus* were collected from the Khulna University campus, Bangladesh. The leaves were thoroughly washed with tap water followed by Milli-Q water to remove dust and adhering impurities, and then air-dried at room temperature for two weeks. The dried leaves were ground into a fine powder using a clean laboratory grinder. To prepare the extract, 5.0 g of the powdered leaf material was mixed with 100 mL of DI water in a 250 mL beaker and heated at 60 °C for 20 min under continuous magnetic stirring at 500 rpm. The suspension was allowed to cool naturally to room temperature and then filtered through Whatman No. 1 filter paper to obtain a clear aqueous extract. The filtrate was stored at 4 °C and used within 48 h as the bio-reductant and stabilizing agent for nanoparticle synthesis.^55^

### Synthesis of IONPs

Magnetic iron oxide nanoparticles were synthesized by a green co-precipitation route using *Phyllanthus acidus* leaf extract as a dual-function reducing and capping agent. In a typical synthesis, 0.33 g of FeCl_3_ and 0.26 g of FeSO_4_·7H_2_O were dissolved in 50 mL of DI water in a 250 mL three-neck round-bottom flask to maintain an Fe^3+^ : Fe^2+^ molar ratio of approximately 2 : 1. The precursor solution was stirred at 600 rpm and heated to 80 °C for 30 min to ensure complete dissolution and homogenization. Subsequently, 5 mL of freshly prepared *Phyllanthus acidus* leaf extract was added dropwise at a rate of approximately 1 mL min^−1^ while maintaining the temperature at 80 °C. The reaction mixture was further stirred for 1 h to facilitate phytochemical-assisted reduction and surface functionalization of the iron species. After this period, 1.0 M NaOH was added dropwise under continuous stirring until the pH reached approximately 10.0, as monitored using a calibrated digital pH meter. The reaction was then continued at 80 °C for an additional 30 min. During this stage, the solution color changed from brown to black, indicating the formation of iron oxide nanoparticles. The resulting black precipitate was separated using an external neodymium magnet, washed several times with DI water until neutral pH was reached, and then rinsed once with ethanol to remove loosely bound organic residues and facilitate drying. To minimize oxidation during purification, washing was carried out under a gentle argon atmosphere. The purified nanoparticles were dried in a vacuum oven at 60 °C for 24 h and stored in an airtight glass vial for further analysis and adsorption experiments.^29,55^

**Fig. 17:**
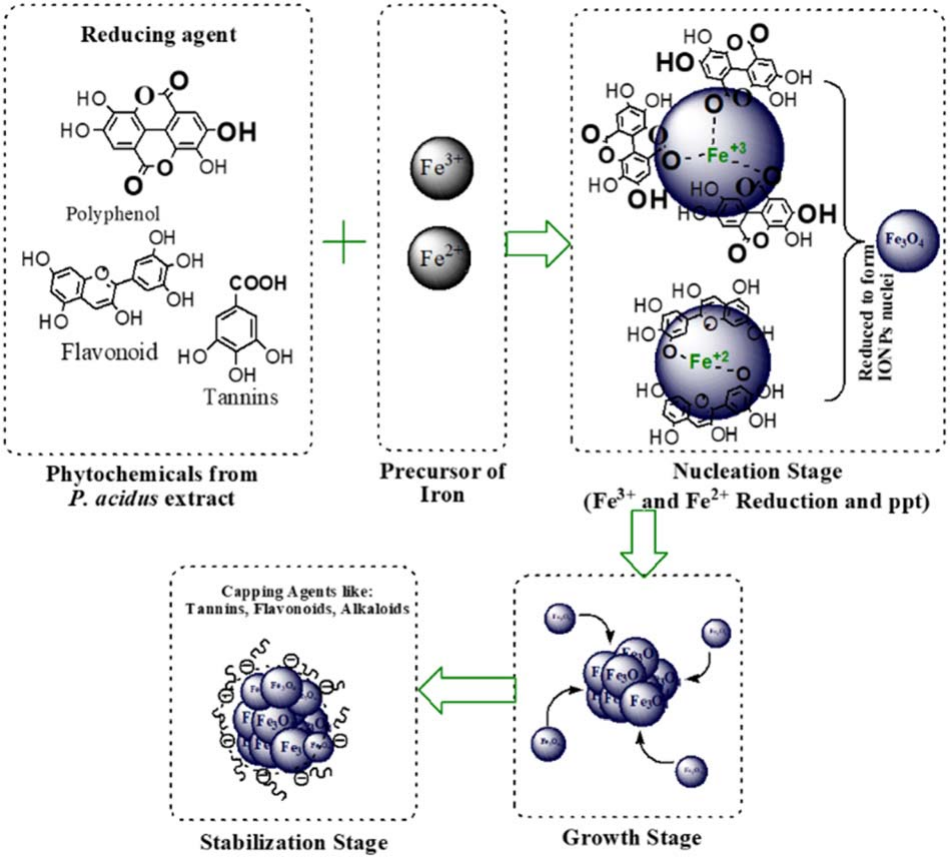
Proposed mechanism for green synthesis of iron oxide nanoparticles mediated by *Phyllanthus acidus* phytochemicals.

Here, the phytochemicals in *P. acidus* leaf extract act as reducing agents to convert Fe^3+^/Fe^2+^ into iron oxide nuclei, while their functional groups simultaneously adsorb onto the nanoparticle surface, regulating nucleation, directing growth, and providing steric stabilization against aggregation (Fig. 17).^56^

### Point of zero charge (pH_PZC_)

The point of zero charge of the synthesized IONPs was determined by the pH drift method. Briefly, 15 mL portions of 0.01 M NaCl solution were transferred into a series of 50 mL polypropylene centrifuge tubes. The initial pH values were adjusted from 2 to 12 using 0.1 M HCl or 0.1 M NaOH. Then, 20 mg of IONPs was added to each tube, and the suspensions were shaken at 150 rpm for 48 h at room temperature. After equilibration, the final pH of each suspension was measured. The difference between the initial and final pH values was plotted as ΔpH = pH_*f*_ − pH_*i*_ against pH_*i*_, and the pH_PZC_ was taken as the pH corresponding to ΔpH = 0. A blank electrolyte series without adsorbent was also maintained under identical conditions to confirm negligible pH drift from atmospheric CO_2_ dissolution.

### Batch adsorption experiments

Batch adsorption experiments were performed at ambient temperature (25 ± 2 °C) in 250 mL beaker containing 100 mL of CR solution. A known mass of IONPs was added to the dye solution, and the suspension was agitated on an orbital shaker at 200 rpm to ensure uniform mass transfer. Operational parameters, including contact time, solution pH, initial dye concentration (5–25 mg L^−1^), and adsorbent dosage (4–15 mg), were systematically optimized. Following the adsorption period, the suspension was centrifuged at 10 000 rpm for 4 min. The residual CR concentration in the supernatant was quantified *via* UV-vis spectrophotometry at *λ*_max_ = 498 nm. The removal efficiency (RE) and the adsorption capacity (*q*_t_) were determined using the following mass balance equations:^57^1
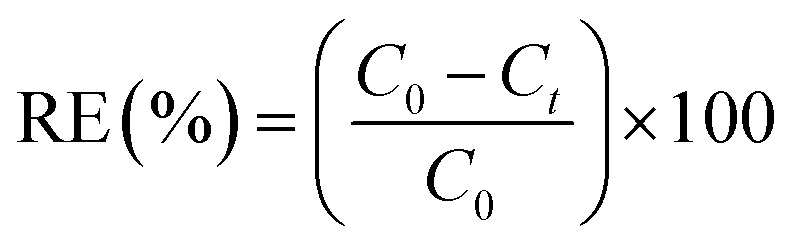
2
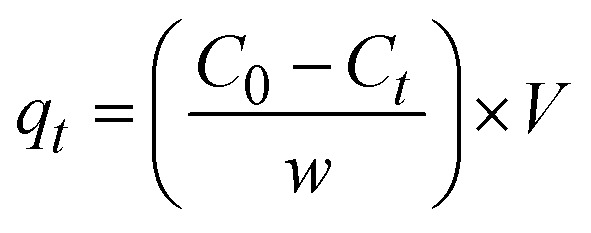
where *C*_0_ and *C*_*t*_ (mg L^−1^) represent the initial and time *t* dye concentrations, respectively; *V* (L) is the volume of the solution, and *w* (g) is the mass of the IONPs.

The effect of pH on CR adsorption was examined over the pH range 2–12. For each experiment, 15 mg of IONPs was added to 100 mL of 25 mg L^−1^ CR solution, and the pH was adjusted using 0.1 M HCl or 0.1 M NaOH before adsorption. The mixtures were shaken at 200 rpm for 3 h to ensure equilibrium. After separation of the adsorbent, the residual CR concentration was measured spectrophotometrically. The solution pH plays a pivotal role in the process, as it governs the surface charge of the IONPs and the ionization state of the anionic CR molecules. Maximum adsorption efficiency was observed at pH 4, as shown in Fig. 8. This performance at slightly acidic conditions suggests enhanced electrostatic attraction between the protonated surface sites of the IONPs and the negatively charged sulfonic groups of the CR dye, consistent with previous literature.

To determine the adsorption rate and equilibrium time, 15 mg of IONPs was added to 100 mL of 25 mg L^−1^ CR solution at pH 4, and the suspension was shaken at 200 rpm. Aliquots were withdrawn at predetermined time intervals of 0, 10, 20, 30, 45, 60, 80, 100, 120, and 130 min. The withdrawn aliquots were immediately centrifuged and analyzed by UV-visible spectroscopy. The adsorption data were fitted to the pseudo-first-order (PFO), pseudo-second-order (PSO), and Weber–Morris intraparticle diffusion (IPD) kinetic models using linearized equations.

The effect of adsorbent dosage was studied by varying the IONP mass from 4 to 15 mg while keeping the CR concentration at 25 mg L^−1^, solution volume at 100 mL, pH at 4, and contact time at 80 min. After equilibrium, the residual dye concentration was measured and both removal efficiency and adsorption capacity were calculated. As expected, the percentage removal increased proportionally with adsorbent dosage due to the increased availability of active binding sites. However, the removal efficiency reached a plateau at higher loadings, indicating site saturation, a characteristic trend in adsorption processes where a fixed number of binding sites are available for a given initial adsorbate concentration. Similarly, the influence of initial dye concentration was investigated from 5–25 mg L^−1^ using a constant 15 mg of adsorbent mass to establish the equilibrium adsorption capacity.

### Adsorption–desorption study

The adsorption–desorption capacity and reusability of the nanoadsorbent were evaluated over five consecutive cycles. In each adsorption phase, 15 mg of the IONPs were introduced into 100 mL of a 25 mg L^−1^ Congo red (CR) solution at an optimized pH of 4. Following adsorption, the IONPs were isolated using an external magnetic field, dried, and analyzed *via* FTIR spectroscopy to confirm the presence of the dye and the structural integrity of the particles. For the desorption phase, the dye-loaded nanoparticles were treated with 0.1 M NaOH at pH 12, utilizing the alkaline environment to facilitate dye release. After separation, the regenerated IONPs were dried and subjected to further FTIR analysis to verify their stability. Precise pH control throughout the experiments was maintained using HCl and NaOH, ensuring consistent conditions across all five cycles.

## Conclusion

This study successfully demonstrated a sustainable, one-pot green synthesis of magnetic iron oxide nanoparticles (IONPs) using *Phyllanthus acidus* leaf extract as an eco-friendly reducing and capping agent. The phyto-fabricated IONPs proved to be an exceptionally potent and recyclable nanoadsorbent for detoxifying Congo red (CR) dye from aqueous systems. The adsorption process was found to be highly pH-dependent, with maximum removal achieved under acidic conditions (pH 4). This behavior is governed by the strong electrostatic attraction between the protonated IONP surface (pH < pHpzc) and the anionic sulfonate groups of the CR molecules. Kinetic modeling revealed that the CR uptake follows a pseudo-second order (PSO) model, indicating that the rate-limiting step is dominated by chemisorption involving electron sharing between the nanoparticle surface and the dye molecules. While intraparticle diffusion contributes to the process, it is not the sole rate-determining factor, pointing toward a complex, multi-step adsorption mechanism. The superparamagnetic properties of the nanoadsorbent enable rapid magnetic recovery, highlighting its potential for cost-effective wastewater treatment. However, further work is needed to evaluate the performance of the synthesized iron oxide nanoparticles (IONPs) in real industrial wastewater containing competing ions and organic pollutants, to better assess their selectivity and robustness under complex conditions. In addition, large-scale synthesis and process optimization should be explored to confirm scalability and economic feasibility. Surface functionalization may further enhance adsorption capacity and broaden the range of target contaminants. Moreover, long-term stability, regeneration over multiple cycles, and potential environmental impacts require systematic evaluation. Overall, *Phyllanthus acidus*-derived IONPs offer a promising and sustainable approach for practical wastewater remediation, in line with the increasing demand for green and efficient environmental technologies.

## Author contributions

Md. Ahad Mahmud Nahim: validation formal analysis and data collection. Asifur Rahman and Jamil Ahmed: writing original draft and writing – review & editing, Jannatul Naime and Md. Abu Rayhan Khan: data analysis and methodology. Shofiur Rahman: writing – review & editing, writing original draft, funding acquisition, validation, investigation, Mahmoud A.-Gawati: data analysis and Habib Md. Ahsan: review & editing, writing – original draft, supervision, funding acquisition methodology, investigation, conceptualization.

## Conflicts of interest

There are no conflicts to declare.

## Data Availability

The datasets generated and/or analyzed during the current study are available from the corresponding author on reasonable request.
